# Targeted Memory Reactivation during Nonrapid Eye Movement Sleep Enhances Neutral, But Not Negative, Components of Memory

**DOI:** 10.1523/ENEURO.0285-23.2024

**Published:** 2024-05-24

**Authors:** Dan Denis, Jessica D. Payne

**Affiliations:** ^1^Department of Psychology, University of York, York YO10 5DD, United Kingdom,; ^2^Department of Psychology, University of Notre Dame, Notre Dame, Indiana 46556

**Keywords:** emotion, memory, reactivation, sleep, sleep spindles

## Abstract

Emotionally salient components of memory are preferentially remembered at the expense of accompanying neutral information. This emotional memory trade-off is enhanced over time, and possibly sleep, through a process of memory consolidation. Sleep is believed to benefit memory through a process of reactivation during nonrapid eye movement sleep (NREM). Here, targeted memory reactivation (TMR) was used to manipulate the reactivation of negative and neutral memories during NREM sleep. Thirty-one male and female participants encoded composite scenes containing either a negative or neutral object superimposed on an always neutral background. During NREM sleep, sounds associated with the scene object were replayed, and memory for object and background components was tested the following morning. We found that TMR during NREM sleep improved memory for neutral, but not negative scene objects. This effect was associated with sleep spindle activity, with a larger spindle response following TMR cues predicting TMR effectiveness for neutral items only. These findings therefore do not suggest a role of NREM memory reactivation in enhancing the emotional memory trade-off across a 12 h period but do align with growing evidence of spindle-mediated memory reactivation in service of neutral declarative memory.

## Significance Statement

Memory reactivation during sleep is believed to be a key mechanism facilitating consolidation, the strengthening and stabilization of memories over time. Emotional memories appear to be preferentially consolidated compared with neutral information, but the role of sleep-related memory reactivation in this process is still unclear. Here, we found that experimentally reactivating memories during sleep in humans did not preferentially enhance emotional memory components but did improve neutral memory when tested after one night of sleep. These findings speak against a role of memory reactivation in the early stages of emotional memory consolidation.

## Introduction

Intrinsically emotional experiences occupy a privileged place in our memory. Emotional events more strongly capture our attention (Yiend, 2010), are remembered more vividly (Kark and Kensinger, 2019), and are more resistant to forgetting compared with neutral information (Yonelinas and Ritchey, 2015). So strong is this effect that emotionally salient aspects of an event are preferentially remembered at the expense of accompanying neutral information (Kensinger et al., 2007). Numerous studies have documented this emotional memory trade-off effect, where negative aspects of scenes are preferentially remembered at the expense of accompanying neutral components, with no such effect being shown for completely neutral scenes (Kensinger et al., 2007; Payne et al., 2008; Waring et al., 2010; Sopp et al., 2018; Denis et al., 2022).

At initial encoding, an array of neurophysiological processes differentiate emotional from neutral stimuli (Phelps and LeDoux, 2005; Shields et al., 2019; Kim and Payne, 2020). Despite these immediate differences, the emotional memory bias becomes magnified over time, as memory performance diverges for emotional and neutral information after a delay of at least a few hours compared with immediate memory testing (Dolcos et al., 2005; Sharot and Yonelinas, 2008; Payne et al., 2012). This suggests that a period of memory consolidation, the process of memory stabilization and strengthening, prioritizes emotionally salient material (Stickgold and Walker, 2013; Kim et al., 2020; Cowan et al., 2021).

Sleep is an optimal state for memory consolidation to occur, as incoming sensory information is diminished (Brodt et al., 2023). A wealth of behavioral evidence shows enhanced memory retention over a period sleep compared with an equivalent amount of time spent awake (see Berres and Erdfelder, 2021 for a comprehensive meta-analysis), even when the amount of wake-associated interference is equated (Talamini et al., 2008; Payne et al., 2012; Denis et al., 2020). Emerging evidence points to a role of memory reactivation during nonrapid eye movement sleep (NREM) underlying sleep's beneficial effect on memory (Klinzing et al., 2019; Denis and Cairney, 2023). During NREM, memories are repeatedly reactivated in hippocampal-neocortical circuits, a process clocked by the temporal coupling of hippocampal sharp-wave ripples (∼80–150 Hz), thalamocortical sleep spindles (∼12–15 Hz), and neocortical slow oscillations (SOs, ∼1 Hz; Latchoumane et al., 2017; Cairney et al., 2018; Zhang et al., 2018; Schreiner et al., 2021; Denis and Cairney, 2023).

A recent framework proposes that these mechanisms of NREM consolidation are biased toward emotional salient information (Cowan et al., 2021). In other words, memory reactivation, coordinated by SO-spindle coupling, should favor the reactivation of emotional memories. The emotional memory trade-off effect appears to be enhanced over a night of sleep compared with a day awake (Payne et al., 2008) an effect that has been frequently replicated (Payne and Kensinger, 2011; Payne et al., 2012, 2015; Cunningham et al., 2014; Alger et al., 2018), including in a recent large-scale study of 280 participants [Denis et al., 2022; though see Bennion et al. (2015, 2017) for notable nonreplications]. These results occur against the backdrop of recent meta-analyses that have been unable to detect such effects when assessing the broader literature, collapsing across experimental designs and task types (Lipinska et al., 2019; Schäfer et al., 2020). Although we note that the idea is speculative, the trade-off effect within complex scenes is more sensitive to the effects of sleep than other commonly used tasks (Davidson et al., 2021), possibly because the task mimics the natural formation of emotional memories more accurately than other tasks such as basic image recognition, or it teases out the separate trajectories different components of a memory may take by allowing us to test objects and backgrounds separately.

Across the existing literature, no consistent correlations between any aspect of sleep and emotional memory have been found (Davidson et al., 2021). With regard to the emotional memory trade-off, some studies have found NREM sleep and sleep spindles to correlate with the magnitude of the trade-off, speaking in favor of NREM reactivation processes (Payne et al., 2015; Alger et al., 2018). On the other hand, a different study found correlations with rapid eye movement sleep (REM; Payne et al., 2012), in line with other research suggesting that REM sleep, and REM theta (4–8 Hz) oscillations in particular, selectively enhance emotional memories (Nishida et al., 2009; Sopp et al., 2017; Kim et al., 2020).

Such studies are correlational in nature and do not directly test the process of reactivation. Targeted memory reactivation (TMR) is an experimental technique through which the reactivation of certain memories can be directly manipulated. In this paradigm, unique sounds are paired with stimuli during encoding, with half being replayed during subsequent sleep. These sounds induce increases in sleep spindle activity and the reinstatement of memory-related content (Bendor and Wilson, 2012; Rothschild et al., 2017; Belal et al., 2018; Cairney et al., 2018; Schreiner et al., 2018; Wang et al., 2019; Ngo and Staresina, 2022). Meta-analytic evidence has shown that memory for items that were reactivated during sleep is enhanced compared with the unreactivated memories (Hu et al., 2020). The application of TMR to the question of emotional memory has yielded mixed results. While some studies suggest that emotional memories do benefit from reactivation in NREM (Cairney et al., 2014; Lehmann et al., 2016; Yuksel et al., 2023), null findings have also been reported (Rihm and Rasch, 2015; Ashton et al., 2018; Hutchison et al., 2021; Pereira et al., 2022). Similar mixed results have been reported when TMR is applied during REM (Sterpenich et al., 2014; Lehmann et al., 2016; Hutchison et al., 2021; Yuksel et al., 2023). However, no studies to date have examined the effect of TMR on the emotional memory trade-off effect (i.e., does TMR enhance emotional information at the expense of accompanying neutral information?).

The present study set out to test the hypothesis that reactivation during NREM sleep facilitates the selective enhancement of negative aspects of memory. By combining the emotional memory trade-off task, which may be more sensitive to sleep effects compared with other tasks (Davidson et al., 2021), and TMR to manipulate memory reactivation, the following specific hypotheses were tested: (1) negative components of memory would be preferentially enhanced at the expense of accompanying neutral components; (2) this emotional memory trade-off effect would be further boosted by TMR during NREM sleep; and (3) the effect of TMR would be associated with TMR-evoked sleep spindle activity. As an exploratory aim, correlational analyses between ongoing oscillatory activity during sleep with emotional memory were performed. These analyses focused on SO-spindle coupling during NREM sleep, based on prior work suggesting oscillatory coupling to be a mechanistic driver of consolidation (Klinzing et al., 2019; Denis and Cairney, 2023), and REM theta oscillations, as a putative marker of REM-based emotional memory consolidation (Hutchison and Rathore, 2015).

## Materials and Methods

### Participants

Participants were 31 undergraduate students recruited at the University of Notre Dame (*M* [95% CI] = 20 [19, 21], 68% female). Inclusion criteria for the study included no self-reported history of any sleep, neurologic, or psychiatric disorders, a self-reported bedtime of no later than 2 A.M. and sleeping on average for no less than 6 h per night. All participants were instructed to keep a regular sleep schedule for the three nights prior to the study and to abstain from caffeine the day of the study. All participants provided informed consent prior to taking part in the study and were compensated with either cash or course credit. A second group of 32 participants, recruited from the same population and with the same inclusion criteria, served as a no TMR control group. They performed the exact same procedure, with the exception that no sounds were played during sleep. Demographic information for both groups can be found in Table 1. Recruitment was through advertisement of the study on SONA and flyers placed around the University of Notre Dame campus. The study received ethical approval from the University of Notre Dame Institutional Review Board.

**Table 1.** Demographics and subjective sleep and alertness measures

	TMR group		No TMR group		*t*/*χ*^2^	*p*
	*M*	CI	*M*	CI		
Age (years)	20	[18, 21]	20	[19, 20]	0.07	0.95
Sex						
Female (%)	68	[51, 84]	53	[36, 70]	0.86	0.35
Male (%)	32	[16, 49]	47	[30, 65]		
PSQI	4.65	[3.84, 5.45]	4.63	[3.54, 5.71]	0.03	0.98
MEQ	45.71	[42.63, 48.78]	45.34	[42.36, 48.32]	0.17	0.86
Sleep log bedtime	01:04	[00:30, 01:38]	01:29	[00:46, 02:12]	0.94	0.36
Sleep log rise time	08:28	[08:08, 08:48]	08:59	[08:19, 09:40]	1.44	0.16
Sleep log TST (mins)	444	[411, 478]	451	[418, 484]	0.29	0.78
Encoding subjective alertness	2.81	[2.35, 3.26]	2.44	[2.07, 2.80]	1.28	0.20
Recognition subjective alertness	3.10	[2.63, 3.54]	2.81	[2.38, 3.25]	0.91	0.37

*M*, mean; CI, 95% confidence interval. Sex expressed as a percentage of participants. PSQI, Pittsburgh Sleep Quality Index. Higher score indicates worse subjective sleep quality (theoretical range, 0–21). MEQ, Morningness-Eveningness Questionnaire. Higher score indicates greater evening preference (theoretical range, 16–86). TST, total sleep time. Sleep log measures are the average of the three nights prior to the experimental night.

### Design

#### Study overview

**Figure 1.**
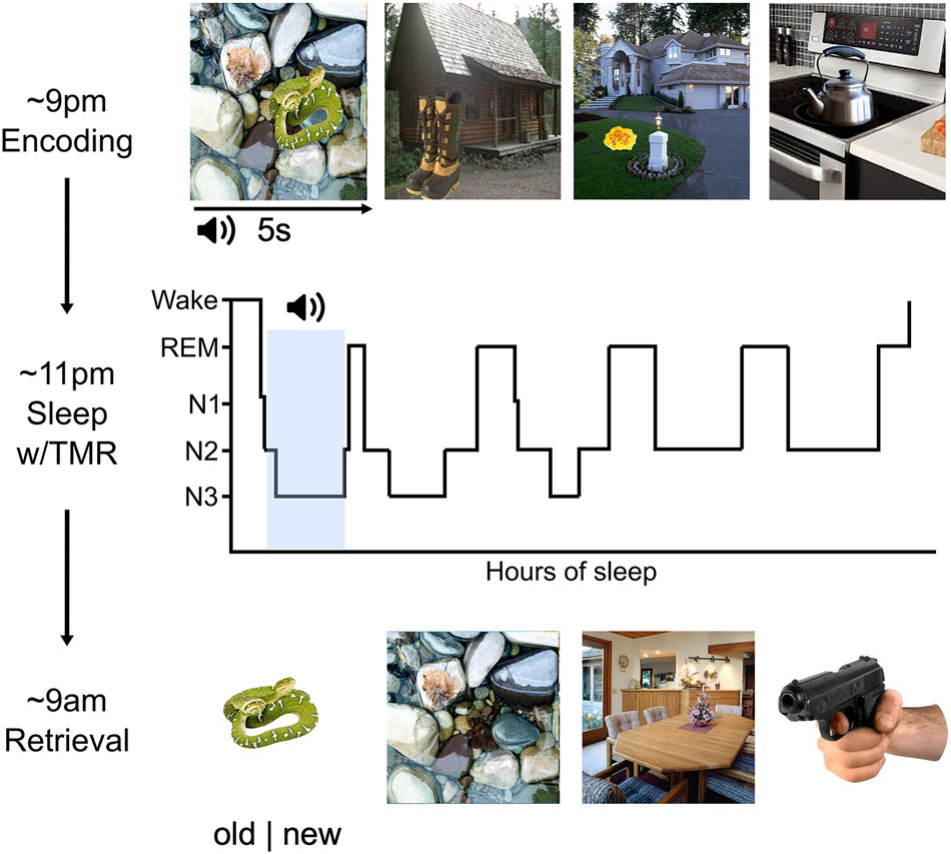
Experimental design. In the evening, participants incidentally encoded 92 scenes containing either a negative or neutral object superimposed on an always neutral background. Each scene was accompanied by a sound naturally linked to the object. During the first hour of nonrapid eye movement sleep, half of the sounds were replayed. A surprise memory test was administered the following morning. Participants viewed scene components individually and had to indicate whether the component was old or new.

The study design is depicted in Figure 1. After providing informed consent, participants filled out questionnaires regarding their subjective sleep habits over the past three nights, general subjective sleep quality over the past month as assessed by the Pittsburgh Sleep Quality Index (Buysse et al., 1989), and their diurnal preference as assessed by the Morningness-Eveningness Questionnaire (Horne and Ostberg, 1976). Following this, participants were wired for EEG (see below). They then reported their subjective levels of alertness via the Stanford Sleepiness Scale (Hoddes et al., 1972), before completing the incidental encoding portion of the emotional memory trade-off task. At ∼11 P.M., participants went to bed and had a 9 h sleep opportunity, before being awoken at 8 A.M. the next day. During the first hour of NREM (N2 + N3) sleep, half of the sounds were replayed (see below, Targeted memory reactivation). No sounds were played in the NO-TMR control group. In the morning, participants had the EEG cap removed and were given the opportunity to shower. They then completed a second Stanford sleepiness scale assessment, before completing the recognition portion of the emotional memory trade-off task. See Table 1 for all subjective sleep and alertness measures.

#### Emotional memory trade-off task

The studied materials consisted of 92 scenes depicting negative (*n* = 46) or neutral (*n* = 46) objects placed on plausible, always neutral backgrounds. Each scene was accompanied by a sound that was conceptually related to the object component of the scene (e.g., a “woof” sound was paired with a scene depicting a dog in a park). The trade-off scenes used were taken from a larger database of scenes and have been used in previous studies (Cunningham et al., 2021; Denis et al., 2022). The sounds were taken from a prior unpublished report (Personal communication, 2019) and from the website Freesound (https://freesound.org/). The sounds were 500 ms in duration (Creery et al., 2015) and were played through a loudspeaker at ∼70 dB (Schreiner et al., 2015).

The emotional memory trade-off task consists of an initial incidental encoding portion followed by a surprise recognition test after the delay (Fig. 1). During encoding, participants viewed each of the 92 scenes in a randomized order. Each trial began with a fixation cross displayed in the center of the screen for 1 s (±200 ms intertrial jitter) before the scene was displayed on the screen for 5 s. The associated sound was played at the onset of each trial. Participants were instructed to rate the scene for its perceived valence and arousal. At the end of each trial, participants rated each scene on a 1–5 scale for its valence (very negative to very positive) and arousal (not at all arousing to very arousing). After a 2 s intertrial interval, the next trial began. Participants were instructed to remain still and relaxed and to limit eyeblinks and movements while the scene was on the screen. There was a break halfway through to allow participants to stretch and adjust their posture. At no point during the encoding session were participants informed that their memory would be tested.

Following the sleep period, participants completed a surprise, self-paced recognition test in which the objects and backgrounds were presented separately and one at a time. The old objects and backgrounds that comprised the full scenes at encoding were intermixed with an equal number of new object and background components of scenes that were not viewed at encoding. For each trial, participants were instructed to indicate whether the component was old or new as quickly and as accurately as possible using the keyboard. There was a total of 368 trials at recognition [92 old objects (46 negative, 46 neutral), 92 old backgrounds (46 originally paired with negative objects, 46 originally paired with neutral objects), 92 new objects (46 negative, 46 neutral), and 92 new backgrounds (all neutral)]. No sounds were played at the recognition test.

#### Targeted memory reactivation

Soft pink noise was played out of a bedside speaker throughout the sleep period at ∼45 dB (Schechtman et al., 2023). When participants reached 5 min of uninterrupted N2 or N3 sleep, cueing ensued. A total of 46 randomly selected sounds (23 negative, 23 neutral) that had been presented with scenes during encoding were played, along with six (three negative, three neutral) controls sounds that were not associated with any of the scenes presented at encoding. All 52 sounds were presented in a random order, with a 5 s inter-stimulus interval. After all 52 sounds were presented, the order was rerandomized and a new cueing block began. Cues were presented during both N2 and N3 sleep and continued for 1 h or until the first period of rapid eye movement sleep was reached. Cueing immediately stopped upon sign of an arousal or shift to N1 sleep or wakefulness. In these cases, cueing resumed after re-entering N2 or N3 sleep.

### EEG

#### Acquisition

EEG was collected from all participants during both the encoding and sleep portions of the experiment at a sampling rate of 500 Hz. For this analysis, we focused on just the sleep EEG recordings. Data were acquired from 58 electrodes positioned according to the 10–20 system. Additional electrodes were placed on the left and right mastoid (for offline rereferencing), above the right and below the left eye (for EOG measurements), and two placed on the chin (for EMG measurements). A BrainVision actiCHamp Amplifier and Recorder software was used to acquire the data. Impedances were kept below 15 kΩM. Data were sleep scored offline according to standard criteria (Iber et al., 2007). Sleep architecture is shown in Table 2.

**Table 2.** Sleep architecture on the experimental night

	TMR group		No TMR group		*t*	*p*
	*M*	CI	*M*	CI		
Total sleep time (min)	503	[492, 515]	476	[459, 494]	2.11	0.04
Sleep onset latency (min)	24.5	[16.18, 32.86]	25.36	[17.05, 33.67]	0.23	0.82
Wake after sleep onset (min)	33.91	[28.32, 39.49]	31.50	[26.09, 36.91]	0.45	0.66
Sleep efficiency (%)	90.62	[88.91, 92.32]	89.23	[87.20, 91.25]	0.75	0.46
N1 time (% of TST)	4.40	[2.76, 6.03]	3.27	[2.68, 3.85]	1.29	0.20
N2 time (% of TST)	54.60	[51.89, 57.30]	55.70	[53.39, 58.00]	0.61	0.55
N3 time (% of TST)	18.50	[16.38, 20.62]	21.01	[19.21, 22.81]	1.77	0.08
REM time (% of TST)	22.51	[20.12, 24.90]	20.03	[17.95, 22.16]	1.53	0.13

*M*, mean; CI, 95% confidence interval; TST, total sleep time.

#### EEG preprocessing

Data were downsampled to 250 Hz. Bad channels were identified by eye and interpolated using a spherical splines algorithm. Next, EEG data were notch filtered at 60 Hz, high-pass filtered at 0.3 Hz, and rereferenced to the average of the two mastoid channels. Data were then epoched from −1 to 3 s around the onset of all cues that were presented during either N2 or N3 sleep. Bad epochs were identified as those exceeding a voltage threshold of ±500 µV, and using the joint probability function in EEGLAB (Delorme and Makeig, 2004), with a threshold of 6 standard deviations for single channels and 2 standard deviations for the global signal (all channels grouped). Finally, each record was visually inspected to ensure adequacy of the artifact rejection procedure.

#### TMR analysis

Clean, artifact-free epochs were baseline adjusted to the mean voltage in the 1 s before cue onset. Complex Morlet wavelets were then used to decompose the time series data into time-frequency representations (TFRs). Spectral power was extracted at 30 logarithmically spaced frequencies from 2 to 40 Hz with the number of wavelet cycles increasing from 5 to 10 in 30 logarithmically spaced steps to match the number of frequency bins. Time-frequency power was averaged over trials, and decibel normalized within-participant, where the baseline was mean power in the 500–200 ms prior to cue onset. This choice of baseline was chosen so as to mitigate contamination of the baseline period by poststimulus activity. As in prior TMR experiments, analyses were performed at electrode Cz (Schechtman et al., 2021).

Sleep spindles that occurred −1 to 3 s around TMR cues were detected using an automated algorithm (see below for details; Antony et al., 2018; Cairney et al., 2018; Schechtman et al., 2021). For each spindle that was detected, its amplitude was extracted. To calculate the probability of a spindle occurring following a TMR cue, established procedures were followed (Schechtman et al., 2021; Liu et al., 2023). For each trial, a spindle value of 1 was assigned to the time points where a spindle was detected and 0 where no spindle was detected. The spindle probability was then computed as the mean spindle values across trials for each time point.

#### Slow oscillation-spindle coupling

SO-spindle coupling was detected at all EEG electrodes during artifact-free NREM sleep using well-validated approaches. First, individual channels where at least one Hjorth parameter was >3 times the standard deviation of the mean for at least 25% of all epochs was considered bad and was interpolated. Second, individual epochs were removed if >50% of channels had at least one Hjorth parameter >3 times the standard deviation of the mean. For epochs where <50% of channels were above the threshold, these channels were interpolated on an epoch-by-epoch basis. For both steps, artifact detection was performed over two iterations (Purcell et al., 2017; Denis et al., 2021a, 2023).

Sleep spindles were detected at all electrodes using a wavelet-based detector (Wamsley et al., 2012; Warby et al., 2014). As a first step, we identified each participant's fast spindle peak frequency from their NREM power spectrum. Power spectral density (PSD) was estimated using Welch's method with 5 s Hamming windows and 50% overlap. PSD estimates were obtained from the derivative of the EEG time series to minimize 1/*f* scaling and maximize spectral peaks (Cox et al., 2017). The largest, most prominent peak within a broadly defined fast spindle range of 12.5–16 Hz was taken as that individual's peak spindle frequency. After detecting each individual's spindle peak frequency, the EEG signal was subject to a time-frequency decomposition using complex Morlet wavelets. The wavelet parameters were tuned for each individual based on their spindle peak. Specifically, the peak frequency of the wavelet was set at that individual's spindle peak, with a 3 Hz bandwidth (full-width, half-max) centered on the peak frequency (Denis et al., 2021b). Spindles were detected by applying a thresholding algorithm to the extracted wavelet scale. A spindle was detected whenever the wavelet signal exceeded a threshold of nine times the median signal amplitude of all artifact-free data for a minimum of 400 ms. The threshold of nine times the median empirically maximizes between class (“spindle” vs “nonspindle”) variance in previous samples of healthy participants with 12–15 Hz overnight spindles (Mylonas et al., 2020).

SOs were detected using a second automated detector (Staresina et al., 2015). First, data were bandpass filtered between 0.5 and 4 Hz and all positive-to-negative zero crossings were identified. Candidate slow oscillations were marked if two such consecutive zero crossings fell 0.8–2 s apart, corresponding to 0.5–1.25 Hz. Peak-to-peak amplitudes for all candidate oscillations were determined, and oscillations in the top quartile (i.e., with the highest amplitudes) at each electrode were retained as SOs (Staresina et al., 2015; Helfrich et al., 2018).

SO-spindle coupling events were identified using a co-occurrence approach. First, EEG data were bandpass filtered in the individualized spindle frequency range. Then, the Hilbert transform was applied to extract the instantaneous phase of the delta (0.5–4 Hz) filtered signal and the instantaneous amplitude of the spindle filtered signal. For each detected spindle, the peak amplitude of that spindle was determined. It was then determined whether the spindle peak occurred within the time course (i.e., between two positive-to-negative zero crossings) of any detected slow oscillation. If the spindle peak was found to occur during a slow oscillation, the phase angle of the slow oscillation event at the peak of the spindle was calculated. We extracted the density of coupled spindles (calculated as the number of slow oscillation-coupled spindles per minute of NREM sleep) and the average coupling phase (in degrees) and consistency (vector length).

#### REM PSD

To estimate REM theta PSD, artifactual REM epochs were removed using the same automated artifact rejection procedure as above. REM PSD estimates were obtained at each EEG channel using the same method as for spindle peak detection. Given that signal amplitude is at least partly driven by individual difference factors such as skull thickness and gyral folding (Cox and Fell, 2020), we then normalized, within participant, each electrode's power spectrum by dividing power at each frequency by that electrode's average power (Denis et al., 2021a). Each individual's peak frequency within a broadly defined theta range (3–8 Hz) was identified from the power spectrum. Theta PSD estimates were then calculated by averaging together PSD in frequencies ranging ±1.5 Hz around the theta peak (Harrington et al., 2020).

### Memory analysis

To assess memory performance, we calculated a corrected recognition score in line with previous research utilizing the emotional memory trade-off task (Cunningham et al., 2021; Denis et al., 2022). For this measure, a “hit” was defined as saying “old” to an old trial, and a false alarm was defined as saying “old” to a new trial. For each scene component, we calculated the proportion of hits and false alarms and then subtracted the false alarm rate from the hit rate to obtain the corrected recognition score. For the purposes of correlating TMR cue-evoked EEG activity with performance, we also calculated the cueing benefit by subtracting corrected recognition scores for the uncued items from corrected recognition scores for the cued items (Groch et al., 2017). As such, a more positive value reflects a greater efficacy of TMR in terms of improving memory.

### Statistical analysis

#### Behavior

Valence and arousal ratings during encoding were analyzed with two separate mixed effects models with emotion (negative, neutral) entered as a fixed effect and participant entered as a random effect. The effect of TMR on the emotional memory trade-off was assessed via a 2 (emotion: negative, neutral) × 2 (component: object, background) × 2 (TMR: cued, uncued) linear mixed effects model with participant entered as a random effect and corrected recognition scores as the dependent variable. Follow-up estimated marginal means tests were used as appropriate, with *p* values adjusted for multiple comparisons using the false discovery rate (FDR). Analyses were performed in R (R Core Team, 2021) using the *lme4* (Bates et al., 2015) and *emmeans* (Lenth, 2022) packages. Statistical significance was established using the *anova* function to obtain the *F* statistic and associated *p* value for each fixed effects term in the model, with *p* values derived using Satterthwaite's degrees of freedom approximation method implemented in the *lmerTest* package (Kuznetsova et al., 2017). For completeness, response times during encoding and recognition were also analyzed using the same mixed effects model framework as the ratings/memory accuracy data. To test whether the cueing benefit was significantly different from zero, we performed one-sample *t* tests on the cueing benefit for each memory component separately. Again, *p* values were adjusted for multiple comparisons using the FDR.

#### EEG

We first contrasted time-frequency responses to memory and control cues by performing paired *t* tests across all time and frequency points. To ensure that any differences between conditions were not due to differences in trial counts, the number of trials was matched between conditions by randomly selecting a subset of trials from the higher trial count condition. A similar approach was used to contrast negative and neutral cues. To control for multiple comparisons across time and frequency points, we employed a cluster-based permutation approach (Maris and Oostenveld, 2007). The cluster statistic (*t*_sum_) was the sum of the test statistics of time-frequency points within the cluster. Permutation distributions were then created by randomly shuffling condition labels 1,000 times at each time-frequency point and retaining the cluster with the maximum statistics for each permutation. The cluster-level corrected *p* value is the probability that the observed cluster would be found by chance under the permutation distribution.

To assess associations between cue-evoked EEG activity and the TMR benefit, time-frequency points in significant clusters were averaged together to obtain a single value for each cluster reflecting memory/emotion-sensitive activity. For each cluster separately, we regressed cueing benefit against cluster power, emotion (Negative, Neutral), and their interaction. Robust regression procedures were used to minimize the influence of outliers.

To examine relationships between endogenous slow oscillation-spindle coupling/REM theta PSD and memory, we also used robust linear regressions. In separate models, we regressed corrected recognition scores against SO-spindle coupling/REM theta PSD at each electrode, with emotion (Negative, Neutral) and the interaction term included as fixed effects. Multiple comparisons across electrodes were controlled for using the same cluster-based permutation testing framework described above. Circular–linear correlations between spindle coupling phase and corrected recognition scores were run at each electrode, with multiple comparisons again being controlled for via cluster-based permutation tests. For coupling phase analysis, negative and neutral memory was analyzed separately.

Differences in oscillatory measures between the TMR and NO-TMR groups were conducted using independent samples *t* tests (Watson–Williams test for coupling phase) at each electrode, with multiple comparisons across electrodes controlled via the same cluster-based permutation framework described above. All EEG analyses were performed in MATLAB, using the FieldTrip (Oostenveld et al., 2011) and circStats (Berens, 2009) toolboxes along with custom code. Robust linear regressions were fitted with the *fitlm* function from MATLAB, with robust options turned on.

## Results

### Behavior

At encoding, participants rated negative scenes as significantly more negatively valanced and significantly more emotionally arousing compared with neutral scenes (valence: negative *M* [95% CI] = 2.57 [2.31, 2.83], neutral *M* [95% CI] = 4.45 [4.23, 4.66]; arousal: negative *M* [95% CI] = 4.49, [4.18, 4.80], neutral *M* [95% CI] = 3.40 [3.16, 3.63]; all *p*s < 0.001). Subjective valence and arousal ratings did not correlate with any aspect of subsequent memory (all *p *> 0.20). There were no differences between negative and neutral scenes in terms of response time for either valence (neutral *M* [95% CI] = 2.07 s [1.91 s, 2.24 s]; negative *M* [95% CI] = 2.13 [2.00 s, 2.67 s]) or arousal (neutral *M* [95% CI] = 2.18 s [2.06 s, 2.29 s], negative *M* [95% CI] = 2.19 s [2.02 s, 2.37 s]) ratings (*p*s > 0.50).

**Figure 2.**
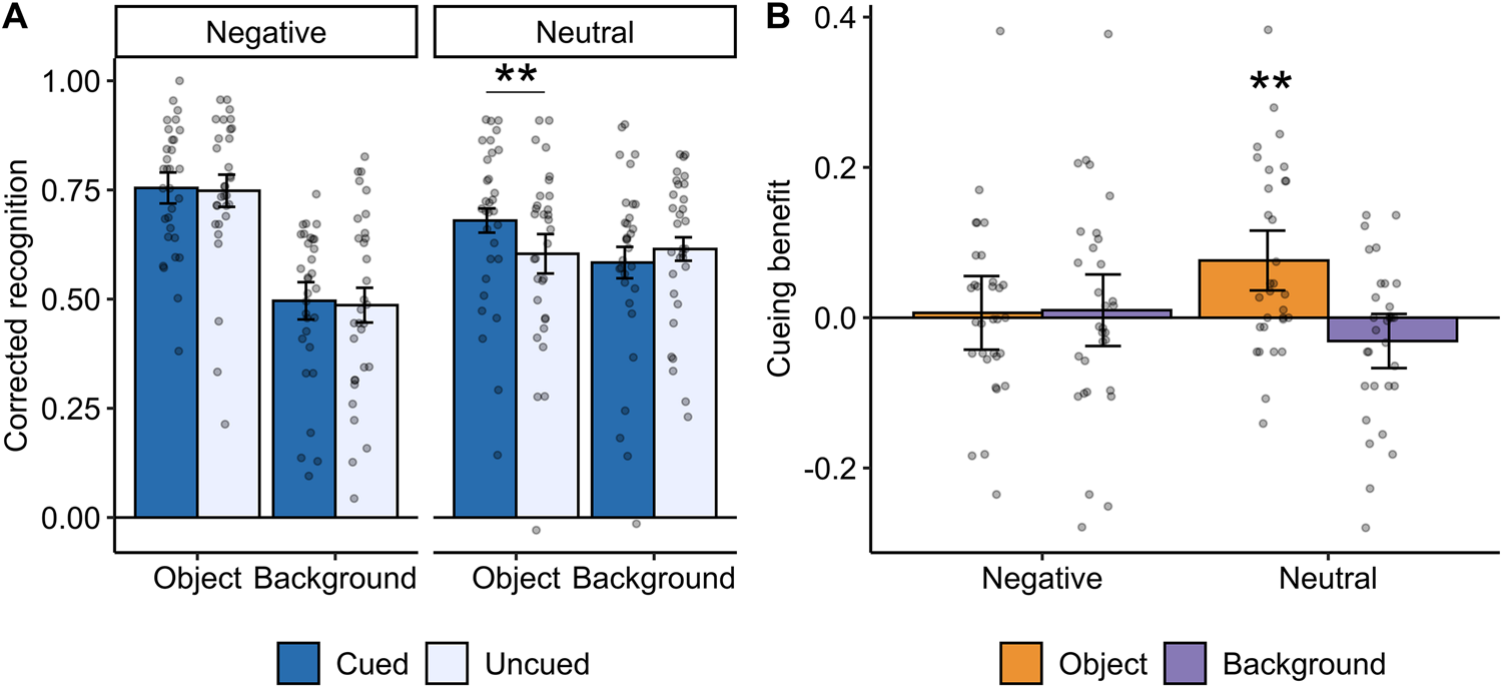
Behavioral results. ***A***, Corrected recognition scores for cued (dark blue) and uncued (light blue) items. **p < 0.01. ***B***, Cueing benefit (cued minus uncued corrected recognition). **p < 0.01 (cueing benefit significantly different from 0). All error bars indicate 95% confidence intervals.

We next examined memory scores at the postsleep recognition test (see Table 3 for raw memory scores). A significant interaction between emotion and component (Estimate = 0.16; *F*_(1,210)_ = 72.86; *p *< 0.001) indicated the presence of an emotional memory trade-off effect (Fig. 2*A*). As expected, memory for negative objects (*M* [95% CI] = 0.75 [0.72, 0.78]) was significantly better than memory for neutral objects (*M* [95% CI] = 0.64 [0.61, 0.67]; *t*_(210)_ = 6.07; *p*_adj _< 0.001). This coincided with significantly worse memory for backgrounds initially paired with a negative object (*M* [95% CI] = 0.49, [0.46, 0.52]) compared with backgrounds initially paired with a neutral object (*M* [95% CI] = 0.60, [0.57, 0.62]); *t*_(210)_ = 6.00; *p*_adj _< 0.001.

**Table 3.** Memory scores

	TMR group				No TMR group	
	Cued		Uncued			
	*M*	CI	*M*	CI	*M*	CI
Hit rate						
Negative objects	0.88	[0.84, 0.92]	0.87	[0.83, 0.91]	0.89	[0.85, 0.92]
Negative backgrounds	0.59	[0.55, 0.63]	0.58	[0.54, 0.62]	0.60	[0.57, 0.63]
Neutral objects	0.77	[0.74, 0.80]	0.69	[0.66, 0.73]	0.75	[0.72, 0.78]
Neutral backgrounds	0.67	[0.64, 0.80]	0.71	[0.67, 0.74]	0.71	[0.69, 0.74]
False alarm rate						
Negative objects	0.12	[0.11, 0.14]			0.14	[0.12, 0.16]
Neutral objects	0.09	[0.08, 0.10]			0.10	[0.08, 0.12]
Backgrounds	0.09	[0.07, 0.11]			0.10	[0.08, 0.12]
Corrected recognition						
Negative objects	0.76	[0.72, 0.79]	0.75	[0.71, 0.79]	0.75	[0.71, 0.78]
Negative backgrounds	0.50	[0.45, 0.54]	0.49	[0.45, 0.53]	0.50	[0.47, 0.53]
Neutral objects	0.68	[0.65, 0.71]	0.60	[0.56, 0.65]	0.65	[0.62, 0.68]
Neutral backgrounds	0.58	[0.55, 0.62]	0.61	[0.59, 0.64]	0.50	[0.47, 0.53]

*M*, mean; CI, 95% confidence interval. Corrected recognition calculated as hit rate—false alarm rate.

The overall emotional memory trade-off was superseded by a significant three-way interaction between TMR, emotion, and component (estimate = 0.11; *F*_(1,210)_ = 4.71; *p *= 0.03), suggesting a selective effect of TMR on memory (Fig. 2*A*). Contrary to our hypothesis, there was no difference in memory between negative objects that were either cued (*M* [95% CI] = 0.76, [0.72, 0.79]) or uncued (*M* [95% CI] = 0.75 [0.71, 0.79]); *t*_(210)_ = 0.25; *p*_adj _= 0.80. On the other hand, memory for neutral objects that were cued during sleep (*M* [95% CI] = 0.68 [0.65, 0.71]) was significantly enhanced relative to uncued neutral objects (*M* [95% CI] = 0.60 [0.56, 0.65]); *t*_(210)_ = 2.99; *p*_adj _= 0.013. TMR did not alter memory for backgrounds paired with either negative or neutral objects (*p*s > 0.44). We note these results remained significant after removal of a potential outlier (neutral memory >1.5 * the interquartile range).

The high level of negative object memory could indicate a ceiling effect, such that no additional TMR benefit could be seen. It may be the case that a benefit of TMR would be seen among participants with lower negative object recognition memory. To test for this, we divided participants via a median split based on uncued negative object memory performance (above median group: *M* [95% CI] = 0.86 [0.83, 0.90]; below median group: *M* [95% CI] = 0.63 [0.54, 0.71]). Importantly, the below median group exhibited negative object memory roughly equivalent to neutral object memory (*M* [95% CI] = 0.60, [0.57, 0.62]), where a significant TMR effect was observed. Despite this, there was no difference in cued versus uncued negative object memory within either of these groups (above median group: *t*_(24.7)_ = 1.02, *p *= 0.32; below median group: *t*_(27.1)_ = 0.91, *p *= 0.37).

The TMR benefit for neutral objects was significantly greater than zero (*t*_(30)_ = 3.40; *p*_adj _= 0.008), with TMR facilitating a 7.6% [95% CI: 3.6%, 11.6%] improvement in memory for neutral objects (Fig. 2*B*). The cueing benefit on neutral objects was significantly higher compared with cued neutral backgrounds (*t*_(30)_ = 4.64; *p *< 0.001), suggesting that TMR primarily benefited the object component of the scene. At the individual level, 19 (61% [95% CI: 42%, 78%]) of 31 participants exhibited a beneficial effect (i.e., >0) of TMR on neutral object memory, which was not significantly greater than 50% (*p* = 0.28). Although TMR significantly improved neutral object memory, memory for cued neutral objects was still significantly worse than memory for negative objects (*t*_(210)_ = 2.93; *p *= 0.004).

It is possible that, rather than enhancing memory for cued items, TMR suppresses or impairs memory for the uncued items. To test for this possibility, we compared memory for the uncued items against a separate sample who underwent the exact same procedure, except no TMR cues were delivered during sleep. There was no main effect of group, nor any interactions involving group (all *p*s > 0.15). As such, uncued memories showed similar to performance to what occurs over a night of normal sleep with no external cueing. A supplementary analysis of reaction times during recognition found a faster response to correctly recalled object components (*M* [95% CI] = 1.75 s [1.55 s, 1.95 s] compared with backgrounds (*M* [95% CI] = 1.97 s [1.84 s, 2.09 s]; *F*_(1,158.3)_ = 8.93; *p *= 0.003. No effects of emotion or TMR were observed for reaction times (*p*s > 0.11).

### Cue-evoked EEG activity during sleep

**Figure 3.**
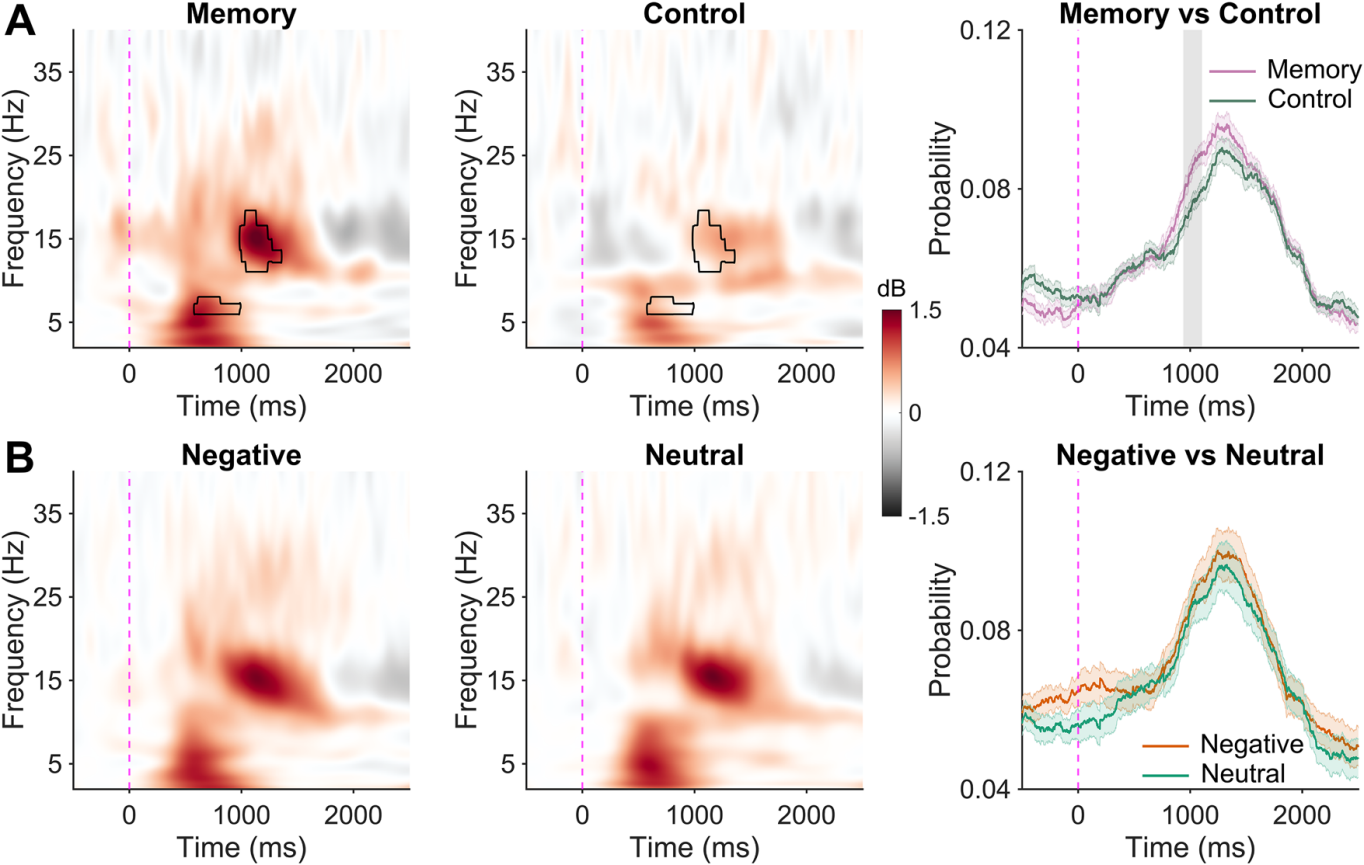
Cue-elicited EEG activity during sleep. ***A***, EEG response to memory and control cues. Time 0 indicates cue onset. Left, Time-frequency representation (TFR) of cue-elicited neural activity following memory-related cues. Middle, TFR of response to control sounds. Significant differences between memory and control cues (cluster corrected) highlighted with black contour. Right, Spindle probability following either memory (pink) or control (green) cues. Significant difference (cluster corrected) highlighted in gray. Shaded area depicts the 95% confidence interval. ***B***, Same as ***A***, but for either negative (left) or neutral (middle) cues. Right, Plot shows spindle probability following either negative (orange) or neutral (green) cues. Shaded area depicts the 95% confidence interval.

We next turned our attention to EEG activity following cue presentation during sleep (number of cues by condition and sleep stage presented in Table 4). We first tested for memory-specific processing by contrasting TFRs to sounds originally paired with a scene during encoding (i.e., memory related cues) to nonlearning control sounds. Memory-related cues evoked significantly larger responses in two time-frequency clusters (Fig. 3*A*). The first cluster emerged 358–1,016 ms following cue onset in canonical theta-band frequencies ranging from 4.57 to 7.65 Hz (*t*_sum _= 3,445; *p *= 0.026). A second cluster formed between 812 and 1,282 ms at frequencies broadly corresponding to the sleep spindle band (14.24–19.41 Hz; *t*_sum _= 1,923; *p *= 0.041). As such, the brain response to sounds cued during sleep was sensitive to whether that sound was associated with a scene viewed during presleep memory encoding.

**Table 4.** Number of sounds presented during sleep

	N2		N3		N2 + N3	
	*M*	CI	*M*	CI	*M*	CI
Negative	81.31	[58.01, 104.61]	165.97	[140.36, 191.58]	247.28	[228.01, 266.56]
Neutral	80.00	[56.73, 103.28]	167.14	[141.43, 192.85]	247.14	[227.88, 266.40]
Control	35.55	[25.06, 46.04]	76.52	[64.66, 88.38]	112.07	[103.07, 121.07]

*M*, mean; CI, 95% confidence interval.

The enhanced power in spindle frequencies could either reflect an increased occurrence of spindles following memory-related cues, higher spindle amplitude following memory cues, or both. Regarding occurrence, there was a significantly higher probability of a spindle occurring following a memory-related cue compared with a control cue 876–1,208 ms following sound onset (*t*_sum _= 605; *p *= 0.008), a time window closely corresponding to cue-evoked increase in spindle power (Fig. 3*A*). On the other hand, there was no difference in spindle amplitude following memory (*M* [95% CI] = 58.24 µV [54.65 µV, 61.83 µV]) or control (*M* [95% CI] = 58.48 µV [54.91, 62.05]) cues (*t*_(58)_ = 0.10; *p *= 0.92).

Having established brain responses specific to memory-related TMR cues, we next examined TFR responses to memory-related negative cues and memory-related neutral cues (Fig. 3*B*). No significant clusters emerged. Similarly, there were no significant differences in either spindle probability or amplitude. As such, it appears that the valence of the cued memory did not impact on the EEG dynamics following sound presentation.

**Figure 4.**
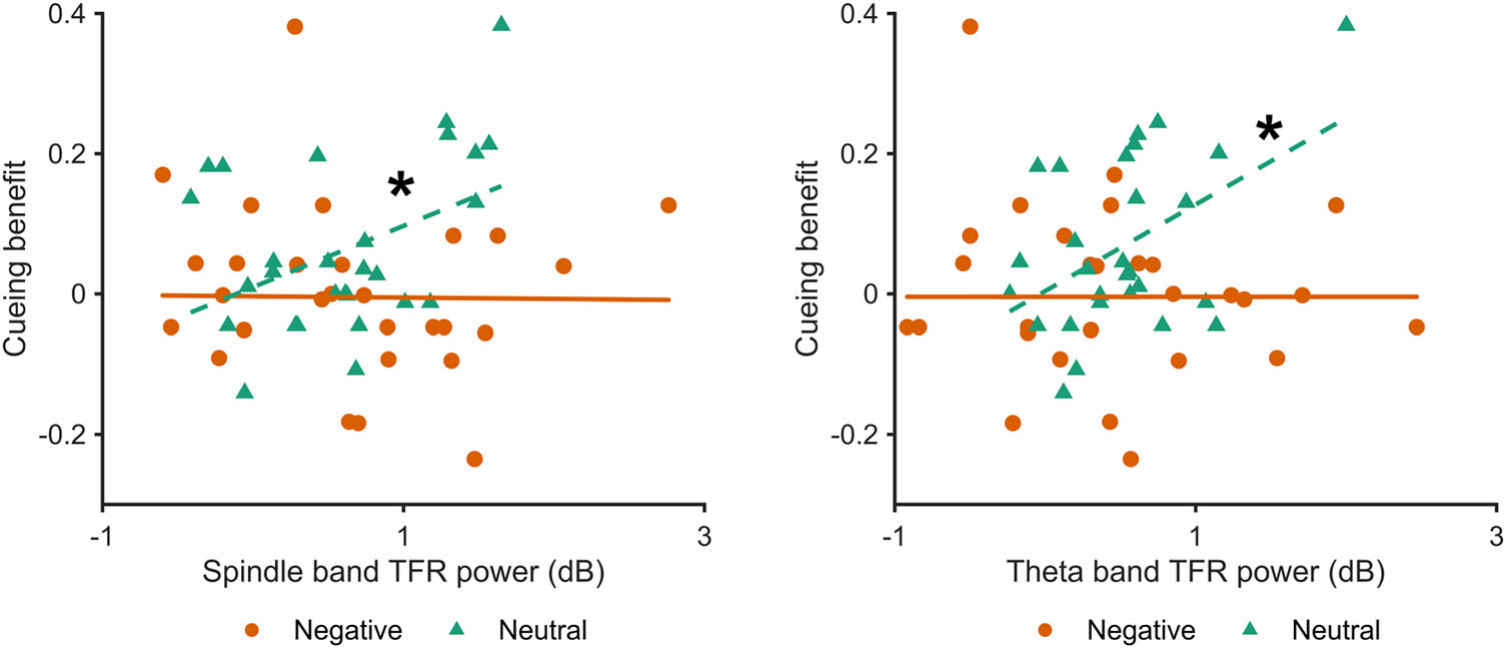
Cue-evoked time-frequency power in the spindle (***A***) and theta (***B***) frequencies bands are associated with cueing benefit of neutral, but not negative, object memory. *p < 0.05.

To test our third hypothesis, we correlated cluster-averaged power in the spindle-band cluster with the benefit of TMR. To isolate a memory-specific signal, we subtracted the control cue response from the memory cue response, separately for negative and neutral cues. Using this measure, there was a significant interaction between spindle band time-frequency power and emotion on the TMR benefit (*F*_(1,52)_ = 4.21; *p *= 0.045; Fig. 4). Cue-evoked spindle power correlated positively with the cueing benefit for neutral objects (*r *= 0.42; *p *= 0.026) but not negative objects (*r *= −0.12; *p *= 0.54). A similar interaction was found for the theta band response (*F*_(1,52)_ = 4.80; *p *= 0.033). Again, cue-evoked theta power correlated positively with the cueing benefit for neutral objects (*r *= 0.46; *p *= 0.014) but not negative objects (*r *= −0.11; *p *= 0.56).

### Endogenous sleep physiology correlations

To examine our exploratory aim, we performed correlational analyses between ongoing, endogenous oscillations believed to facilitate consolidation and object memory. For all measures assessed (spindle density, coupled spindle density, spindle coupling phase and consistency, REM theta power), no main effects or interactions with emotion on memory were found (*p*s > 0.11). Additionally, no main effects or interactions were observed for N3 time, NREM time (N2 + N3), or REM time (*p*s > 0.12). There were no differences between the TMR and NO-TMR group in any measure (*p*s > 0.35).

## Discussion

This study tested the role of memory reactivation during NREM sleep on enhancing negative components of memory. Although we observed a substantial emotional memory trade-off, memory reactivation did not enhance this trade-off further. Instead, reactivation improved memory for neutral objects, an effect mediated by sleep spindle activity. These results further support findings that neutral declarative memories are reactivated and consolidated during NREM sleep. We extend the existing literature by showing that these same processes do not appear to enhance emotional memories across a 12 h delay. We consider several potential reasons as to why TMR did not enhance the emotional memory trade-off effect.

First, it is possible that overnight enhancement of the trade-off is REM-dependent, which would be in line with a prior report utilizing the same task in an overnight design (Payne et al., 2012) and other work implicating REM theta spectral power in selective emotional memory processing (Hutchison and Rathore, 2015). We ruled out an effect of REM sleep in the present study, with no correlations between either REM time or REM theta power being found with emotional memory. This null effect agrees with the larger literature that has not found consistent links between REM sleep and emotional memory (Davidson et al., 2021).

A second explanation could be that because emotional memories were prioritized for consolidation (Kim and Payne, 2020; Cowan et al., 2021), these memories were being reactivated endogenously, with no additive effect of additional reactivations via TMR. This could explain why neutral memories were benefitted by TMR, as these memories would not have been reactivated as often endogenously. Memory reactivation during NREM sleep is closely tied with SO-spindle coupling events (Schreiner et al., 2021); therefore, under this explanation, it would be expected that ongoing SO-spindle coupling would positively correlate with emotional memory. However, no such associations were seen in the present study.

Memory consolidation is a slow process that continues in the weeks and months following encoding (Dudai, 2012). Sleep's impact on emotional memory can be seen years after the original experience (Wagner et al., 2006). Given how powerful the effect of emotion is, it is possible that the benefits of memory reactivation on emotional memory might not be visible after the 12 h delay employed in this study. Future studies should seek to examine whether memory reactivation makes emotional memories more resistant to forgetting over longer intervals. A related explanation is that participants were at a functional ceiling with regard to negative object memory, limiting the capacity to which TMR could further enhance these memories. As well as increasing the length of delay, a higher number of items during encoding could also serve to weaken memory, which may heighten the observed TMR benefits (Creery et al., 2015; Cairney et al., 2016).

Because negative objects were remembered significantly better than neutral objects, it is not possible in this dataset to disentangle the effects of emotion from the effects of memory strength. This is important, because prior research has shown both the benefit of TMR and the consolidating effect of sleep spindles, to be strongest for weakly encoded information (Creery et al., 2015; Cairney et al., 2016; Denis et al., 2021b). Emotional experiences are inherently remembered more vividly than neutral events; however, future research could attempt to better equate memory strength between emotional and neutral memories. This could be achieved via shorter encoding time for emotional versus neutral stimuli or, as suggested above, increasing the number of emotional stimuli to be encoded relative to neutral stimuli.

One implication of our findings is that TMR can be used to enhance otherwise deprioritized memories (i.e., neutral relative to negative memories). A similar finding was reported by Oudiette and colleagues who found that TMR improved memory for low reward memories, but a natural bias toward high reward memories was not boosted by TMR (Oudiette et al., 2013). Together, these findings show that TMR is able to increase the capacity of sleep-associated memory consolidation, presumably through eliciting memory reactivations that would not have occurred endogenously. An intriguing clinical application would be to investigate whether TMR can be used to boost memories that are maladaptively deprioritized, such as positive information in major depression (Talamini and Juan, 2020; Everaert et al., 2022).

Some limitations of the work should be considered. Although we did not find any correlations with REM sleep, we did not include an REM TMR group. We focused on NREM reactivation here for three reasons: (1) a large evidence base already exists for an effect of NREM reactivation on memory consolidation in general (Klinzing et al., 2019); (2) recent models of adaptive memory processing during sleep have placed a clear emphasis on NREM sleep processes (Cowan et al., 2021), and (3) prior TMR studies of emotional memory have found no consistent benefit of reactivating during REM, with one study even finding it induces forgetting of emotional memories (Yuksel et al., 2023). A second limitation is that the relatively low number of trials and high levels of performance for negative items precluded analyses of subsequently remembered versus subsequently forgotten trials, which may have revealed further insights into how memories were being reprocessed following TMR. Finally, although the sounds were conceptually related to the scene objects, we did not test participant's memory for scene–sound pairings. Therefore, it is unclear how well participants associated each sound to individual scenes. Despite this, the fact that TMR did have a behavioral effect (on neutral items) does suggest that the scene–sound pairings were learned to a strong enough degree that replaying the sound during sleep was able to reactivate the accompanying memory trace.

Taken together, the current study provides further evidence that memory reactivation during sleep improves memory for neutral declarative information, an effect mediated by sleep spindle activity. This process does not appear to support the same function for emotional memories over the first 12 h following encoding. This study is the first to attempt to enhance the emotional memory trade-off via a sleep manipulation, and future research should now seek to better understand how memory reactivation during sleep may support this trade-off over longer time scales.

## Synthesis

Reviewing Editor: Christoph Michel, Universite de Geneve Decisions are customarily a result of the Reviewing Editor and the peer reviewers coming together and discussing their recommendations until a consensus is reached. When revisions are invited, a fact-based synthesis statement explaining their decision and outlining what is needed to prepare a revision will be listed below. The following reviewer(s) agreed to reveal their identity: Alison Montagrin. Note: If this manuscript was transferred from JNeurosci and a decision was made to accept the manuscript without peer review, a brief statement to this effect will instead be what is listed below.

The paper has been evaluated by one of the reviewers of the initial submission to JN. While the reviewer considers the work relevant for the field, he/she has some concerns regarding the inadequate reporting of statistics within the methodology, coupled with some unclear aspects.

The detailed comments are appended below. They should be considered in the revision of the manuscript.

Reviewers comments:

he study investigates how targeted memory reactivation (TMR) during non-rapid eye movement (NREM) sleep influences emotional and neutral memory consolidation. Participants encoded scenes with negative or neutral objects superposed on neutral backgrounds, with associated sounds played back during sleep. TMR improved memory for neutral but not negative objects, which was linked to sleep spindle activity. The findings suggest that spindle-mediated memory reactivation plays a role in enhancing neutral declarative memory rather than influencing the emotional memory trade-off over time.

The study is interesting and well-executed, and the findings make a significant contribution to the field of emotional memory and sleep. While significant improvements have been made in response to previous reviewers' comments, there could still be room for further improvement.

My primary concern is that statistics are not adequately reported within the methodology.

1. You did not specify the software used to perform the Linear Mixed Model in the behavioral analyses. Furthermore, it is unclear which statistical test you used to examine the valence and arousal dimensions.

2. While you mentioned the Linear Mixed Model estimates, they were not reported.

3. Did you account for multiple comparisons (e.g., with Tukey adjustments)?

4. For memory scores (and in general), it is preferable to present the Standard Error of the Mean (SEM) or Confidence Interval (CI) rather than the standard deviation (SD). These measures provide a more accurate indication of data variability and improve understanding of the estimate's precision.

5. I would appreciate it if you could provide a table of corrected recognition scores, which were used in the behavioral analyses.

6. Did you investigate reaction times during the memory task or at encoding? Previous studies has found that increased attention to emotional stimuli does not necessarily influence emotional memory when compared to neutral stimuli. Unlike the observed effects on reaction times, memory performance for salient food stimuli did not improve overall when encoded after exposure to emotional sounds (https://www.tandfonline.com/doi/full/10.1080/02699931.2012.722527; https://www.ncbi.nlm.nih.gov/pmc/articles/PMC7781156/ ).

7. In lines 130-131, please refer to Table 1 and include the 1.3 SD on line 131 (instead of 1). It is unclear to me if the control group was analyzed only for the EEG data? In Table 1, could you provide statistics for the two groups separately (control TMR with no sounds and the TMR with sounds), as well as for other relevant tables (if it has been calculated).

8. Was the volume of the sound adjusted for each participant when the stimuli were presented during the encoding process? Was it reduced during the sleep target memory reactivation phase? Were the stimuli presented randomly during encoding?

9. Figures 2A and 2B should clearly indicate which conditions were significant. For example, in Figure 2B, you could draw a solid line between the two conditions and indicate it with an asterisk.
